# London Dispersion as a Design Element in Molecular Catalysis

**DOI:** 10.1021/jacs.5c09212

**Published:** 2025-08-11

**Authors:** Marvin H. J. Domanski, Michael Fuhrmann, Peter R. Schreiner

**Affiliations:** Institute of Organic Chemistry, 9175 Justus Liebig University, Heinrich-Buff-Ring 17, 35392 Giessen, Germany

## Abstract

This Perspective describes the role of London dispersion (LD) interactions as a key factor controlling chemical selectivity. LD arises from the correlated motion of electrons, leading to subtle yet significant stabilization (“steric attraction”), and counterbalances, together with other noncovalent interactions, Pauli exchange repulsion (“steric hindrance”). While chemists have largely relied on the latter to rationalize selectivities in catalyzed reactions, we emphasize here the role LD plays as a key design element in chemical reactions, in particular, for catalyst development.

## Graphical abstract

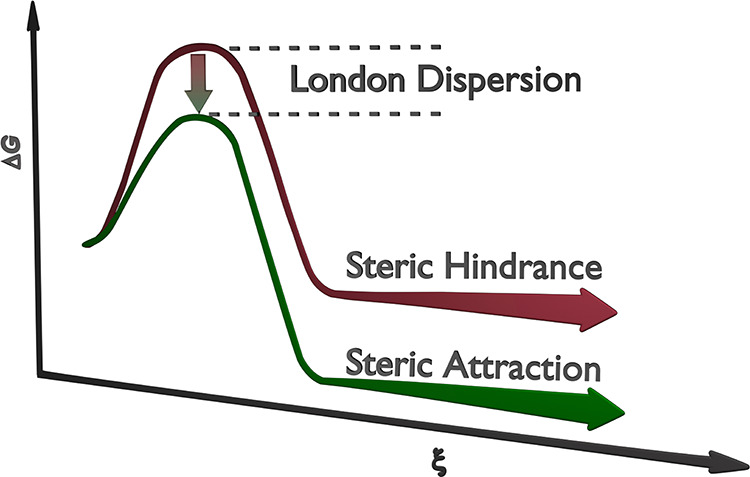

## Introduction

London dispersion (LD)^1^ is a fundamental quantum mechanical phenomenon arising from electron correlation in atoms and molecules. A common “visualization” is to think of LD as induced electron polarization resulting in induced dipoles, leading to attractive interactions that are universally present across all matter.^2^ Hence, LD is a ground-state, time-independent electron correlation phenomenon.^2^

The theoretical foundation for LD was first established by London and Eisenschitz in 1930.^1,3^ Their work provided a mathematical description of these interactions, demonstrating that the dispersion energy follows an *r* ^–6^ dependence, which is now commonly represented by eq 1.^4–9^

1 $$E_{\mathrm{disp}}=-\frac{C_{6}}{r^{6}}$$

*C*_6_, the dipole–dipole dispersion coefficient, is determined by the polarizability α and ionization energy *I* of the interacting partners. The negative sign highlights the attractive nature of LD, with its strength rapidly decreasing as distance *r* increases. Larger system sizes exhibit greater polarizability α (Me 2.6 Å^3^ < Et 4.5 Å^3^ < *n*Pr 6.3 Å^3^ < *n*Bu 8.2 Å^3^ < Ph 10.0 Å^3^) and therefore interact more strongly via LD.^10,11^ This has led to the notion of dispersion energy donors (and mutual acceptors) which follow this trend.^12–14^

In the past decade, LD has been increasingly recognized but not yet used as a routine design element to control chemical reactivity, in particular, for catalyst design. One reason may be that, in contrast to approximately formulated Pauli repulsion (eq 2), LD has no analogue that would reflect human experience, and it is much easier to conceptualize steric repulsion rather than steric attraction. Pauli repulsion is always positive (thus repulsive) and generally much stronger at short distances due to its *r* ^–12^ dependence.^15–18^

2 $$E_{\mathrm{pauli}}=\frac{C_{12}}{r^{12}}$$

These factors likely explain why chemists have traditionally relied on steric arguments–such as those in the Cram model where the stereochemical outcome of nucleophilic addition to carbonyls is exclusively explained via steric repulsion (predominantly of alkyl groups).^19^ However, Pauli repulsion only dominates at very short distances, typically below ∼2.5 Å for hydrocarbons. Beyond that, its influence declines rapidly, and LD becomes the prevailing interaction.^20^ This shift in relative importance is visualized in the characteristic shape of the simplified van der Waals-potential shown in Figure 1 ^17^ and is the very reason why we discuss equilibrium structures.

**Figure 1:**
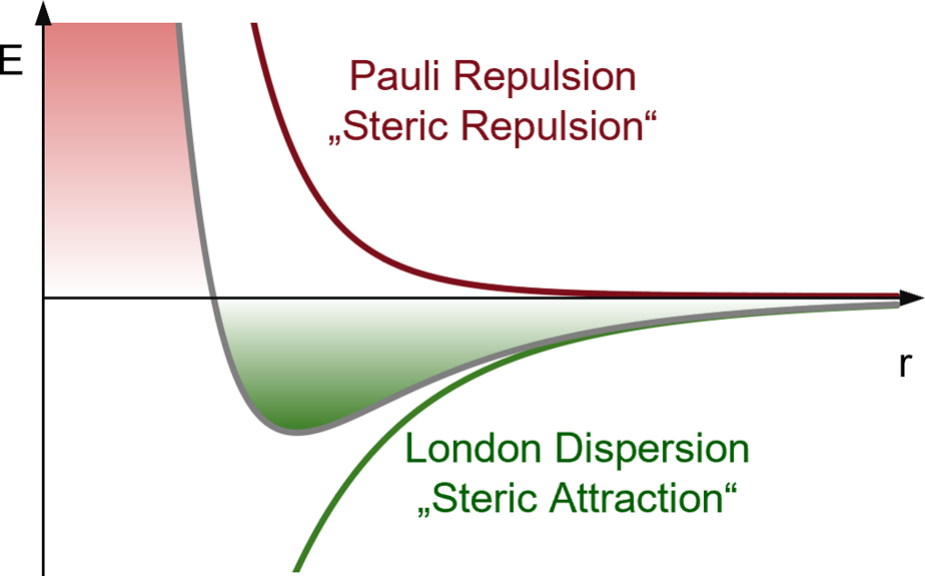
Schematic illustration between LD (green) and Pauli repulsion (red) depending on distance *r*. Resulting binding potential curve in gray.

While London’s insights were groundbreaking, the significance of LD interactions remained largely overlooked for many decades. These interactions were often dismissed as “too weak” to influence molecular behavior meaningfully. Moreover, many practicing chemists argued that such weak interactions would not be relevant in solution because solute–solvent interactions should override such seemingly faint forces. This dismissal is especially surprising, given that LD helps explain fundamental chemical properties–such as the increasing boiling points, the difference in isomerization energies of linear and branched alkanes,^21^ as well as π–π stacking structures in graphite and graphene and the attraction between σ and π systems.^22–24^

In computational chemistry the early implementations of density functional theory (DFT) also dismissed the dispersion component^25^ and it was to a good part the correction of this deficiency^26–30^ that paved the way to analyze the role LD actually plays. Hence, the implementation of LD drastically improved the accuracy of affordable DFT and other approaches to be able to study increasingly larger molecules^31,32^ (for which LD becomes increasingly relevant, *vide supra*), and put the proper physics back into the theoretical modeling.^33,34^ Dispersion-corrected quantum chemical methods fall into three broad categories: (i) empirical corrections (DFT-D*n*), where atom-pairwise dispersion terms are added to conventional DFT energies;^33,35^ (ii) nonlocal functionals (vdW-DF), which incorporate dispersion directly into the exchange-correlation functional via nonlocal correlation terms;^36^ (iii) density-based methods (many-body dispersion),^37^ which derive dispersion interactions from the electron density and atomic properties.^38^ These approaches differ in cost, accuracy, and applicability,^37,39^ but all share the goal of restoring missing long-range correlation to otherwise dispersion-uncorrected functionals.^40^ Additionally, tools became available to visualize LD and other noncovalent interactions (NCIs).^41^ We refer the reader to reviews on this topic for further information.^42,43^

LD has been gaining much more attention in chemistry.^13,14^ For example, molecular balances are being employed to quantify dispersion interactions in solution,^44^ and LD is increasingly considered in catalysis,^14^ where minor changes in relative transition state energies can significantly change the outcome of a reaction. This Perspective aims to introduce the reader to the current concepts, highlights some key examples, and showcases why LD must be considered as an essential design element for catalysis.

## London Dispersion Persists in Solution

Molecular balances can be designed to probe LD interactions^44^ through two thermodynamically accessible and spectroscopically discernible states: a “folded” state, where the interacting groups are in close proximity and an “unfolded” state, where they are spatially separated, thereby preventing their direct interaction (Figure 2). The equilibrium between these states often provides a good measure of the interaction strength, with stronger NCIs shifting the balance toward the folded state.^45,46^

**Figure 2:**
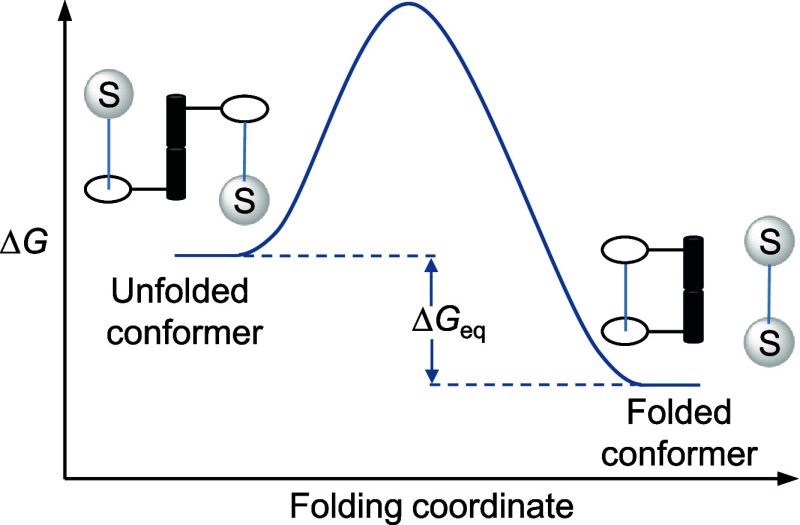
Concept of a two-state molecular torsion balance.^44^

Among the various designs, torsional balances have been particularly useful in isolating and quantifying LD interactions and some recent reviews offer comprehensive overviews of molecular torsion balances.^44,46,47^ Intramolecular LD is typically stronger in the gas phase as other competing interactions are minimized. In solution additional interactions arise that can compete with LD interactions. Chen and co-workers demonstrated this by studying substituted pyridines **1, 2**, and their proton-bound dimers **3** in both the gas phase and dichloromethane (CH_2_Cl_2_) solution (Figure 3).^48^ They found that LD interactions in CH_2_Cl_2_ solution were approximately 70% weaker than in the gas phase.^48^ Follow-up studies expanded this view and included additional polar and nonpolar aprotic solvents.^49^

**Figure 3:**
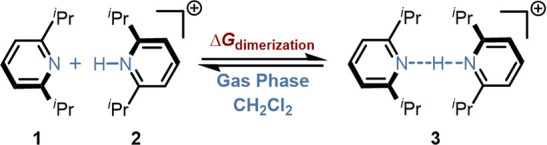
Interaction of pyridine-based proton dimers to investigate LD in the gas phase and in CH_2_Cl_2_ solution.^48^

Cockroft and co-workers investigated how solvent interactions influence LD in polyaromatic stacking.^50^ While gas-phase computations at ωB97X-D/def2-TZVP predicted significant stabilization (up to −6.4 kcal mol^–1^) of the folded conformer **4**_**fold**_ by LD, solution-phase experiments consistently favored the unfolded structure **4**_**unfold**_ (Figure 4). The smallest stabilization of **4**_**fold**_ in solution was observed for substituents such as R = 1-naphthyl (**4–1-Np**), with a measured Gibbs free energy of Δ*G*_fold/unfold_ = +1.1 kcal mol^–1^. The population of **4**_**fold**_ in solution increases with the size of R, whose volume and ability for LD increases concomitantly. Pyrene increases the ratio of the closed conformer **4**_**fold**_ through LD by 1.0 kcal mol^–1^ to a total Gibbs free energy of Δ*G*_fold/unfold_ = +0.1 kcal mol^–1^. In addition, the authors found a correlation between the degree of folding and the bulk solvent polarizability (*P*). Solvents with high bulk solvent polarizability, maximized with a solvent like CS_2_, showed a reduced preference for **4**_**fold**_, while solvents with low polarizability, like CH_2_Cl_2_, enhanced the population of the folded conformer **4**_**fold**_.^50^

**Figure 4:**
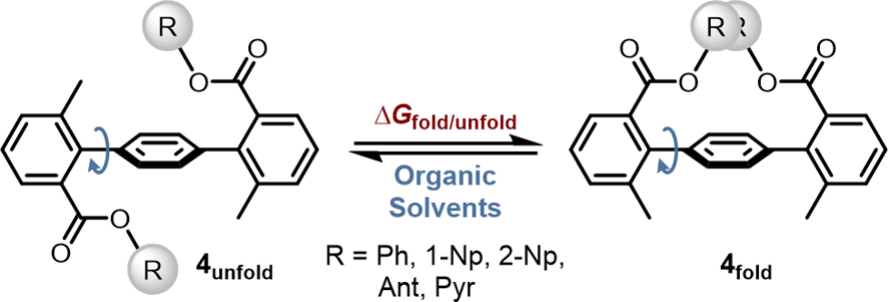
Molecular torsion balance to quantify stacking interactions between polyaromatic residues in solution.^50^

One limitation of these balances is that they are substituted with heteroatoms, leading to local bond dipoles that will have, *inter alia*,^20^ Coulombic interactions with the solvent; separating these from LD can be quite challenging. This situation can largely be avoided by using hydrocarbon molecular balances such as the equilibrium between **1,4-** (unfolded) and **1,6-**(folded) cyclooctatetraene (**COT**) (Figure 5).^51–53^ This system revealed that the attractive interactions between two *t*butyl groups amount to about 0.2 kcal mol^–1^ (Δ*G*) and are largely *independent* of solvent.^54^

**Figure 5:**
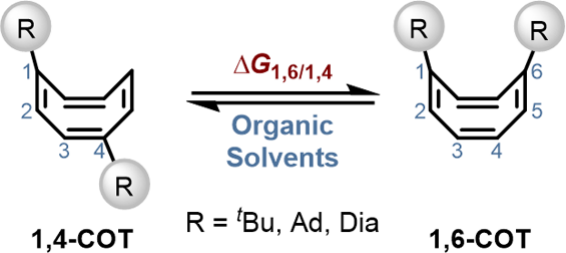
Equilibrium between **1,4**- and **1,6-COT** as a molecular balance to quantify LD interactions in solution.^54,155^

LD interactions are in a first approximation pairwise additive; that is, a single group is not limited to interacting with just one partner but engages with its entire molecular environment at various distances, which all have to be considered. Hence, LD grows rapidly with system size: for example, the interaction of two all-*meta-t*butyl-phenyl residues can be as strong as 1.7 kcal mol^–1^.^55^ Considering that a kinetically controlled catalyzed reaction with a transition state energy difference of 1.7 kcal mol^–1^ gives a ratio of 18:1 at room temperature, this is a non-negligible quantity.

A striking example of the additive nature of LD is found in the prime example of all-*meta-t*butyl-hexaphenylethane. In 1900, Gomberg attempted to synthesize parent hexaphenylethane via the dimerization of two trityl radicals (the first reported organic radical at the time), but the highly symmetric hexaphenylethane *S*_6_-structure proved unattainable.^56–61^ The argument for this instability was the apparent phenyl–phenyl repulsion. Notwithstanding this reasoning, Kahr et al. demonstrated the unexpected stability of all-*meta-t*butyl-hexaphenylethane for which they could even provide an X-ray structure analysis.^62^ The question why the parent molecule does not exist – because it is sterically too hindered– while a closely related derivative that is obviously much more sterically crowded does exist, had apparently not been in focus at the time. Decades later a theoretical study suggested that the all-*meta-t*butyl groups actually are responsible for the stability of this highly crowded Gomberg system through mutually attractive intramolecular LD interactions (Figure 6).^61^

**Figure 6:**
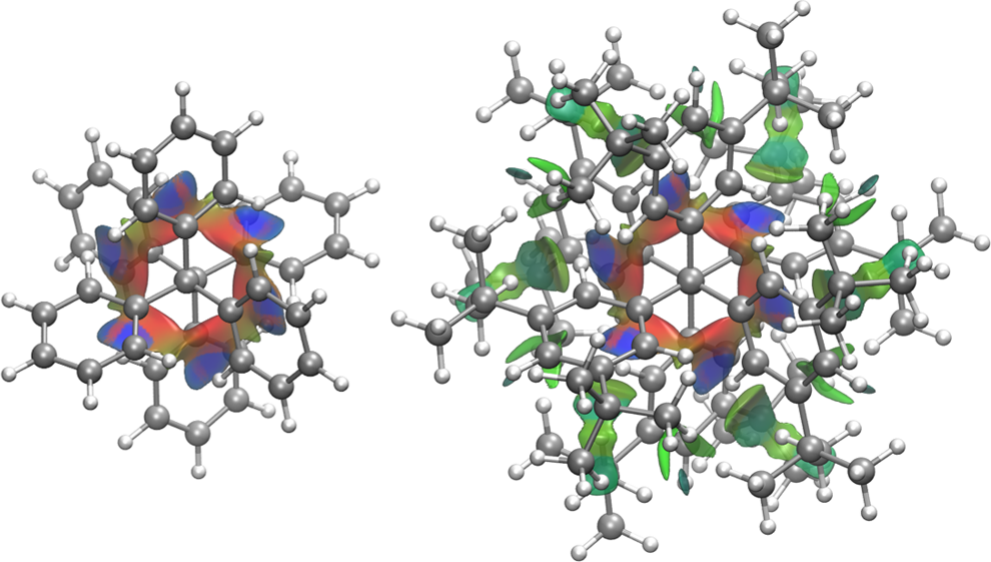
NCI plots^41^ of unsubstituted hexaphenylethane (left) and the *t*butyl substituted derivative (right) viewed along the central carbon–carbon bond.^63^ Blue isosurfaces show strong attraction, red isosurfaces show repulsion, while the substituted derivative benefits from stabilizing LD contacts (green isosurfaces).

NMR spectroscopic experiments confirmed this finding by observing all-*meta*-*t*butyl-hexaphenylethane also in solution.^63–65^ Counterintuitively, the bulky substituents are responsible for the stability of this molecules despite high steric encumbrance. This conclusion is supported by the incorporation of even larger groups in the *meta*-positions such as adamantyl, which amplify LD interactions, leading to even higher stabilization.^64^

## London Dispersion is a Stereochemical Determinant

The use of LD as a stereochemical determinant is under active investigation. In noncatalytic processes, such as the [4 + 2] cycloaddition of benzynes to furans^66^ and the [2 + 2] cyclodimerization of substituted benzynes **5** and **6** (Figure 7),^67^ LD significantly impacts regioselectivity. In both cases, the proximal product **7**, where two substituents are on one side, benefits from LD stabilization relative to the distal product **8**. With weak DEDs such as methyl groups, the [2 + 2] cyclodimerization provides a 1.1:1 ratio, only slightly favoring the proximal product **7**. As the DED strength increases (via size and polarizability),^68^ the product ratio changes in favor of the proximal (more hindered) product **7**. *t*Butyl groups increase the proximal product **7** ratio to 16:1 and adamantyl groups even to >20:1.^67^

**Figure 7:**
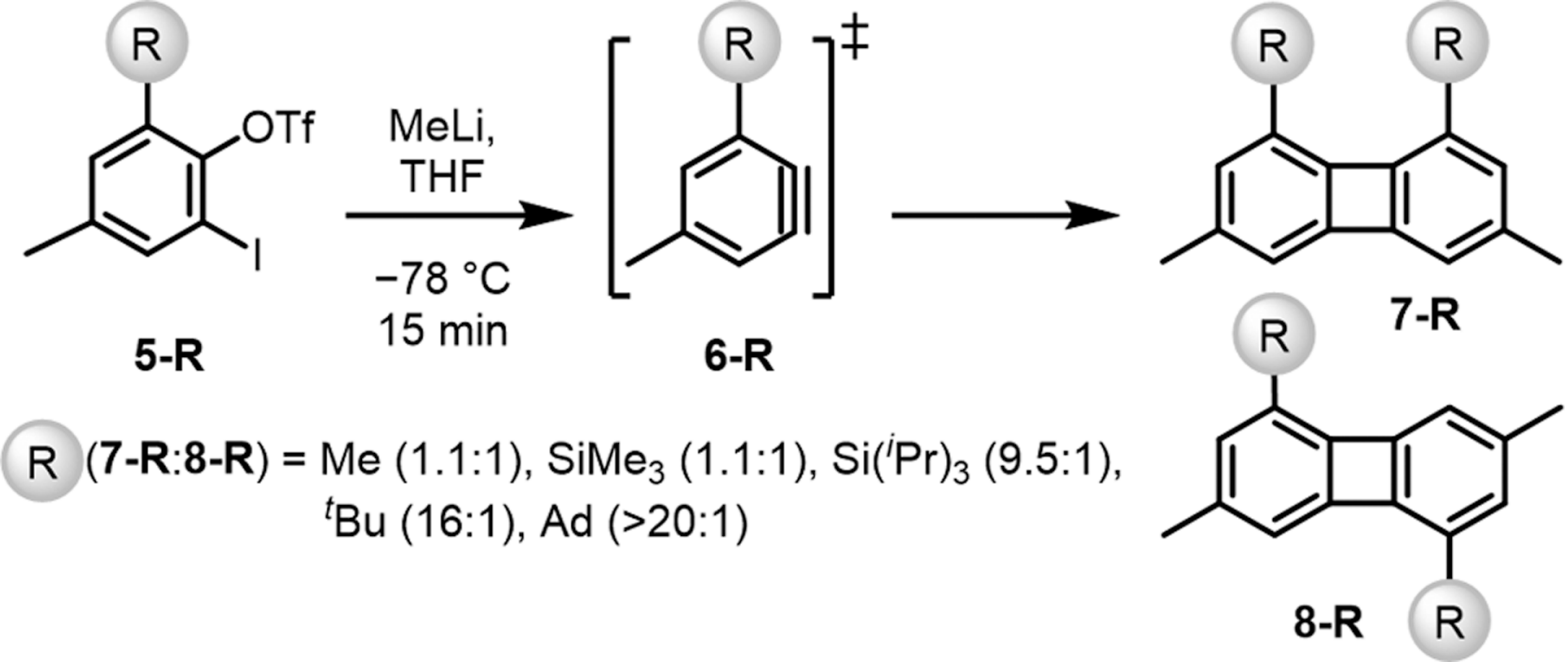
[2 + 2] Cyclodimerization of substituted benzynes. The proximal product is stabilized by LD interaction between the R groups. The selectivity of the reaction increases with the ability of R functioning as stronger dispersion energy donors (DEDs) with increasing size and polarizability (Me < *t*Bu < Ad).^67^

All stereoselective catalyzed reactions must be executed under kinetic conditions because a nonequilibrium product mixture is desired. The difference in energy of the corresponding transition states is responsible for the product distribution and hence stereoselection. Lowering one transition structure, for example, through LD, will significantly change the product distribution even in nonequilibrium processes.^69,70^ For example, the direct impact of LD on a transition structure was demonstrated with the mechanism and selectivities of photochemical dearomative cycloadditions of quinolines **9** with alkenes (Figure 8).^71^ After reversible radical addition, the reaction proceeds through a selectivity-determining radical recombination, favoring the *endo* product **11**. While methyl substituents **11a** lower the transition state energy by 0.7 kcal mol^–1^, *t*butyl groups **11b** increase this value to 1.8 kcal mol^–1^, which the authors attributed to LD.^71^

**Figure 8:**
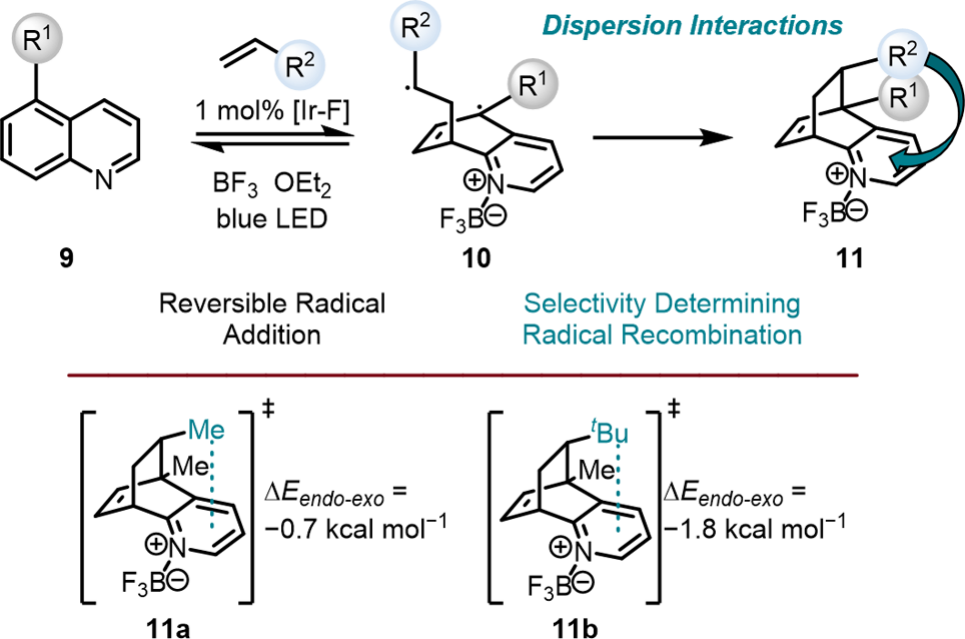
Schematic dearomative cycloaddition of quinolines and alkenes (top). Suggested transition structure of the radical recombination for the *endo* product (bottom).^71^

## Steric Attraction Not Steric Hindrance Makes a Better Catalyst

Unfortunately, LD is still often neglected in the structure–activity relationships between catalysts and substrates.^13^ However, it is essential to consider *all* NCIs, repulsive and attractive, to achieve a meaningful and physically sound understanding of a catalytic mechanism to design better catalysts.^72–77^

With the motivation to demonstrate the importance of LD in catalysis we elaborated on the versatile Corey–Bakshi–Shibata (CBS) reduction of prochiral ketones with oxazaborolidine-based catalysts achieving high selectivity and high yields (Figure 9).^78–83^ The mechanism proposed by Corey in 1992 assumes that stereocontrol arises *exclusively* through steric repulsion between the substituent R on the catalyst **14** and a small R_s_ as well as a large R_L_ on the ketone substrate. In this Cram-based model, the enantiodiscrimination is suggested to originate from a boat-like transition state in which the smaller substituent R_s_ faces R on boron to minimize steric repulsion, while the sterically demanding R_L_ points in the opposite direction. However, several mechanistic findings indicate that Corey’s model is incomplete and cannot deliver a satisfying explanation for the observed selectivities in several reductions; it is also not suitable to rationally improve the catalyst structure. For example, trichloroacetophenone reacts to the corresponding (*R*)-alcohol with high enantioselectivity, which indicates that the large phenyl group faces the boron substituent R. Furthermore, the reduction of cyclopropyl isopropyl ketone, bearing two substituents of very similar steric size, provided an *ee* of 91% in favor of the *R*-enantiomer.^82^ The same holds true for the reduction of *p*-methoxy-*p*′-nitrobenzophenone, where again two substituents of similar size provide an *ee* of 81%.^82^ Hence, steric repulsion alone cannot be taken as a rationale for the observed enantioselectivities.

**Figure 9:**
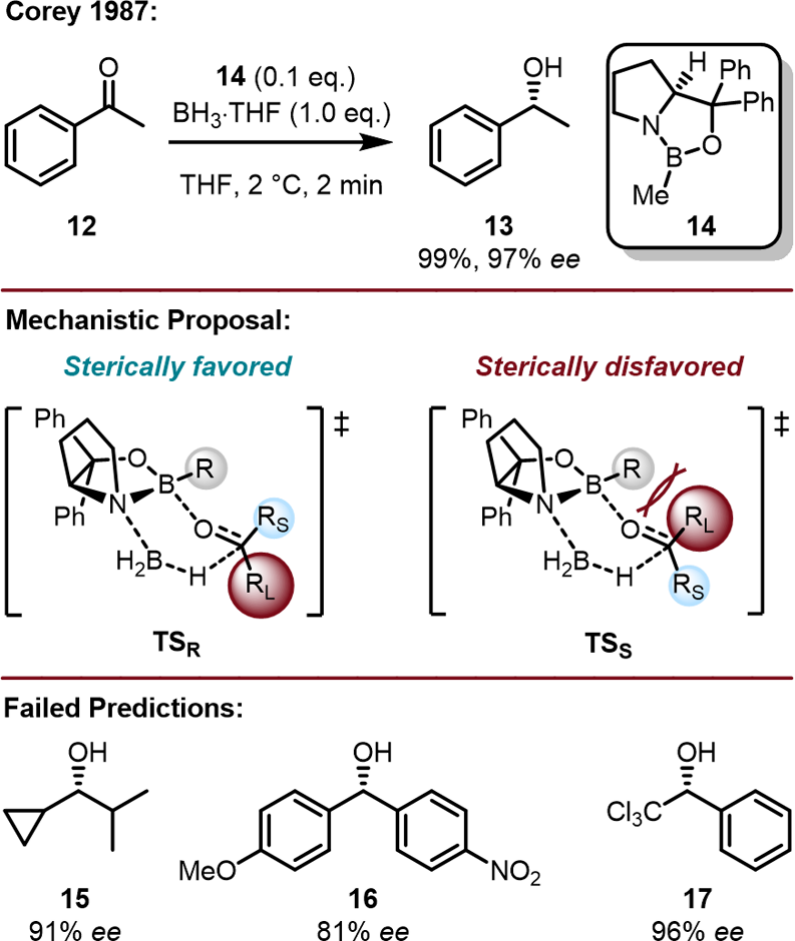
CBS reduction of acetophenone (top), proposed mechanism for the CBS reduction (middle), and selectivities of CBS reductions Corey’s model failed to predict (bottom).^78–83^

Early computational work by Liotta et al. utilizing the MNDO semiempirical approach indicated that the hydride transfer in the CBS reduction proceeds in a chairlike TS rather than the initially proposed boat conformation.^84^ Therefore, the phenyl substituents on the carbinol backbone align parallel with R_L_ to minimize steric repulsion. Liotta’s results are further supported by Meyer investigating the role of steric repulsion in the transition structure of the borane reduction step via kinetic isotope effects.^85^ He observed only a slight contribution of steric repulsion when bulky ketones were employed, which manifests itself in inverse ^2^H kinetic isotope effects. However, this requires the reduction of acetophenone to proceed via the chairlike TS in which the boron substituent R only exerts little influence on the transition structure.^86,87^ A more recent theoretical study by Lachtar et al. demonstrated a more diversified view of NCIs influencing the TS of the oxazaborolidine catalyzed reduction of ketimines.^88^ However, the employed B3LYP/6–31G­(d,p) computations do not include LD corrections and thus neglect major parts of these key NCIs when replacing phenyl groups of the catalyst by hydrogen.

Our findings (Figure 10) demonstrate the importance of LD in understanding the mechanism of the CBS reduction by employing LD and solvent corrections at the B3LYP-D3­(BJ)/6–311G­(d,p)­SMD­(THF) level of theory.^91^ First, the computationally obtained LD-corrected free energies of 13.7 kcal mol^–1^ for **TS1R** and 15.7 kcal mol^–1^ for **TS1S** match the experimental finding that the reaction proceeds at room temperature, in contrast to the energies of 29.8 kcal mol^–1^ and 31.7 kcal mol^–1^, respectively, without the LD correction. The DLPNO–CCSD­(T)/cc-pVTZ single-point energies using DFT-optimized **TS1R** and **TS1S** geometries are provided in brackets for comparison and underline the importance of LD. Furthermore, the computed free energy of the hydride transfer (−2.0 kcal mol^–1^) is consistent with previously determined experimental ΔΔ*G* ^⧧^ (−2.2 kcal mol^–1^).^79^ Note that the **TS1R** and **TS1S** geometries adopt chairlike conformations that are 3.8 kcal mol^–1^ lower in energy than the boat-like transition states initially proposed by Corey et al. Here, the lone pair of the ketone binds to the catalyst that faces the *smaller* substituent R_s_ in an *anti*-fashion to the electron-rich substituent as described by Corey. However, steric hydrogen–hydrogen repulsion is *not* discernible in the NCI plot analysis. On the other hand, stabilizing σ–π LD interactions favor **TS1R**. Decomposing the interaction energies of both transition structures into physically meaningful components with the help of symmetry-adapted perturbation theory (SAPT)^89,90^ analysis further underlines these findings.

**Figure 10:**
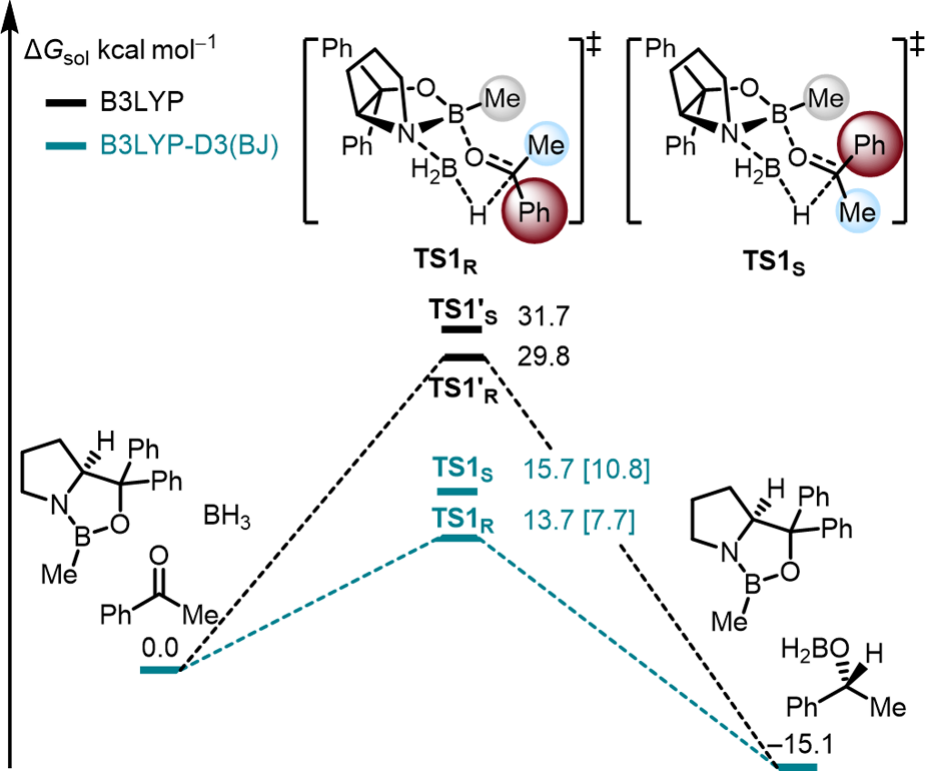
Potential energy surface of the CBS reduction of acetophenone at 2 °C. Free energies (Δ*G*_275K_) computed at B3LYP-D3­(BJ)/6–311+G­(d,p)-SMD­(THF)//B3LYP-D3­(BJ)/6–311G­(d,p). Pathways with (teal) and without (black) dispersion correction. Energies in brackets are based on DLPNO–CCSD­(T)/cc-pVTZ single-point energies (corrected for DFT-ZPVE).^91^

Our experimental results strongly support the computations.^91^ For example, electron-deficient and poorly polarizable substituents (3,5-CF_3_–C_6_H_6_ and C_6_F_6_) on the carbinol backbone gave very low *ee* or even racemic product mixtures in the reduction of acetophenone resulting from reduced LD interactions between substrate and catalyst. On the contrary, bulky and highly polarizable phenyl substituents on the catalyst maximize the *ee*s in the acetophenone reduction. More importantly, for the most challenging substrate butanone, the *ee*s increase with increasing DED-polarizability (Figure 11). Note that all catalysts depicted in Figure 11 perform *better* than those initially proposed by Corey et al. Competitive reduction experiments employing 3,3-dimethylbutan-2-one (pinacolone) and 2-pentanone counterintuitively show faster conversion of the sterically *more* hindered neopentyl substrate. This clearly demonstrates that increased steric bulk does not hamper the reaction rates, in fact, the opposite is the case: the reaction proceeds faster when DEDs are implemented in catalyst and substrate.^91^

**Figure 11:**
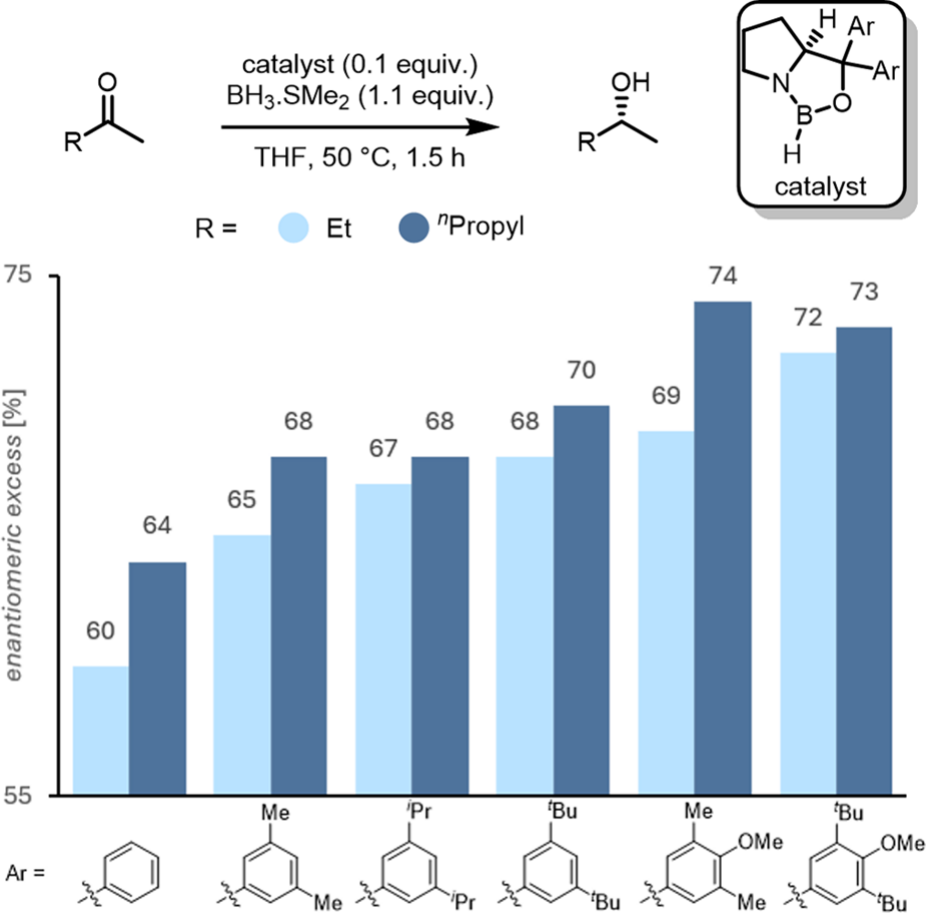
CBS reduction of butanone and 2-pentanone employing various DED-bearing catalysts.^91^

Even the well-established Houk–List model^92^ for understanding the stereochemical outcome of proline catalyzed inter- and intramolecular aldol, Mannich, and Michael reactions, among others, had to be refined after taking into account LD interactions.^93^ The D3 correction at B3LYP/6–31G­(d), initially used by Houk et al.,^92^ changes the relative energies of the stereochemistry-determining transition structures by up to 3 kcal mol^–1^ when benzaldehyde or isobutanal are employed in the proline-catalyzed aldol reaction with cyclohexanone (Figure 12).^93^ Four different stereochemical possibilities arise from the corresponding diastereomeric TSs, with the *anti*-configuration being the energetically most preferred leading to the experimentally observed (*S*,*R*) aldol product **26**. For the reaction with benzaldehyde, the D3 correction between the energetically lowest (*S*,*R*) and (*S*,*S*) TSs accounts for 2.3 kcal mol^–1^ and a ΔΔ*G*_298K_^⧧^ between both competing TSs of 3 kcal mol^–1^ resulting in a population of the (*S*,*R*) isomer of 99.3% (B3LYP-D3/TZVP/SCRF = DMSO) reproducing the experimentally observed *ee* values. This strikingly large energy difference may come as a surprise but impressively demonstrates how large the impact of LD interactions even for small molecules or groups can be. That is, the Houk–List model can lead to large quantitative deviations and, in some cases, fails qualitatively as is the case in the proline-catalyzed reaction of acetone and pyrrole-2-carboxaldehyde. Strikingly, the inclusion of LD turns the initially computed 96% *ee* into an almost racemic product mixture prediction.^93^

**Figure 12:**
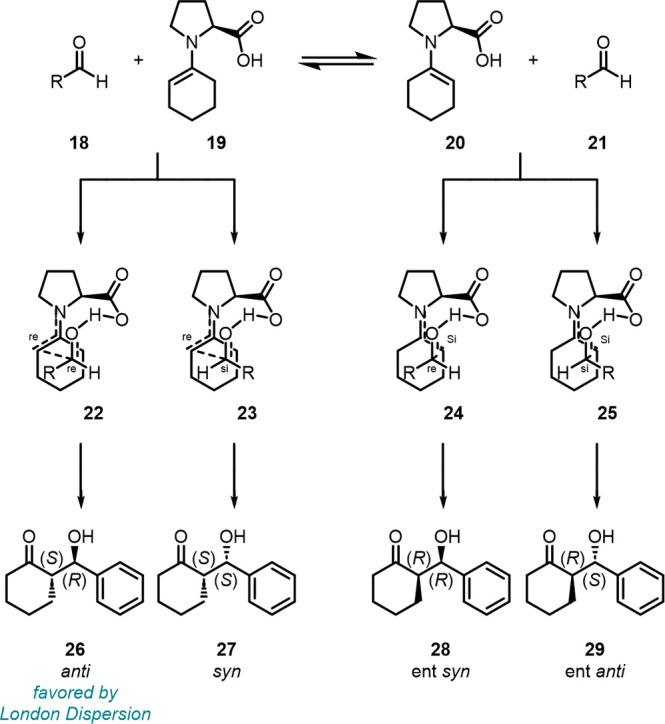
Houk–List model for the proline catalyzed aldol reaction of cyclohexanone with aldehydes.^92,93^

Similarly, a preliminary analysis was put forth by the MacMillan group when they first introduced phenylalanine-derived imidazolidinone in the first highly enantioselective organocatalytic Diels–Alder reaction of iminium ions with dienes in 2000 (Figure 13).^94^ Rationalizing their remarkable results, the group argued that the selective formation of the (*E*)-iminium isomer **30** arises from avoiding steric clashes between the olefin substrate and the geminal methyl substituents on the imidazolidinone and the benzyl group of the catalyst framework sterically shielding the *re* face of the iminium dienophile. Therefore, only the *si* face was suggested to remain exposed for the cycloaddition. In 2004, the Houk group^95^ supported this proposal computationally through investigating alkylation reactions of pyrroles developed by the MacMillan group^197^ with B3LYP/6–31G­(d) computations, which, however, do not account for LD interactions and lack polarization of the hydrogen atoms. This widely accepted MacMillan–Houk model was challenged by Grimme^98^ comparing 14 X-ray crystal structures^96,97^ with LD-corrected DFT (*e.g*., B3LYP-D/def-QZVP-gf) geometries.^98^ The computed relative energies with and without LD correction revealed a difference in stabilization energies of 2.6 kcal mol^–1^ in favor of the (+)-*sc* conformation in which the benzyl moiety faces the geminal *methyl groups* on the catalyst backbone, in line with X-ray crystal structure^96,97^ data of the corresponding iminium salts of the catalysts. The computed conformational energies between (+)-*sc* and (−)-*sc* vary in a small range of ± 2.0 kcal mol^–1^ for various typical substituents in the C(2) position of the heterocycle, which lies in the energetic span of the LD interactions. The benzyl group provides the steric shielding of the iminium π-system via a “*windshield-wiper*” effect freely rotating at ambient temperatures due to the low rotational barriers between the conformers. Therefore, it is necessary to account for LD in the computational model.^40,98^

**Figure 13:**
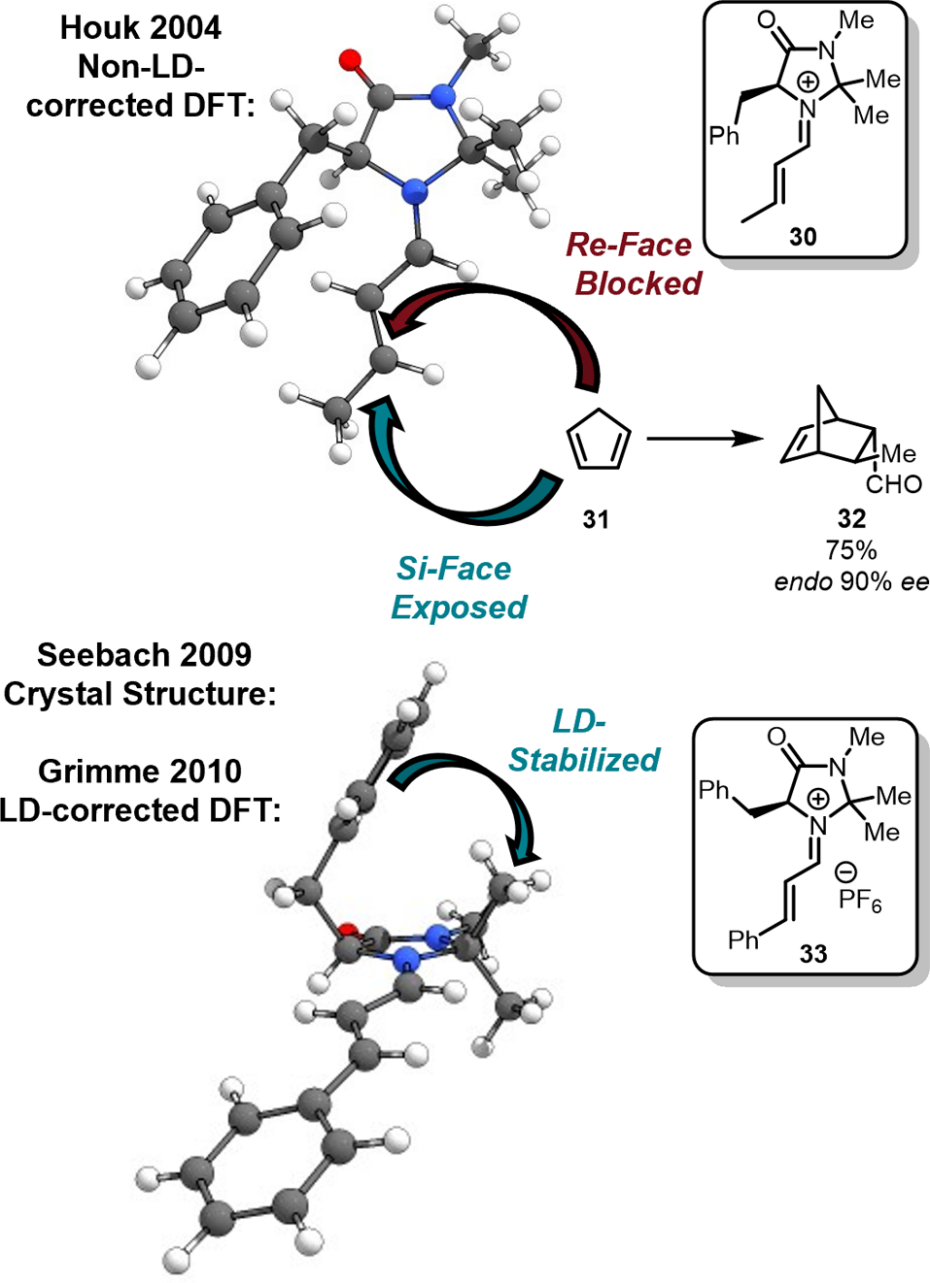
MacMillan’s imidazolidinone catalyst in the initially proposed (−)-*sc* (top)^94–197^ and the LD corrected (+)-*sc* (bottom)^96–98^ conformation.

## Dispersion Energy Donors Enhance Reactivity and Selectivity

Incorporation of bulky aliphatic or aromatic moieties in catalytic motifs has been good practice for decades under the assumption that these groups are sterically active in the sense that they “shield” parts of the interaction space from reacting. The notion of these groups being sterically attractive in their functions as DEDs, however, has only scarcely been recognized. Since the pioneering work of Akiyama^99^ and Terada^100^ in 2004 on chiral phosphoric acid catalysts (CPAs), chemists regularly implemented highly substituted aromatic substituents in 3,3′-position of the BINOL backbone to achieve high enantioselectivities. Well-known examples include MacMillan’s–Si­(Ph)_3_ ^101^ and List’s TRIP^102,103^ substituted CPAs providing exceptional enantioselectivities in reductive transfer hydrogenations of imines, enals, and enones. But how can these catalysts be so general even though they are used in quite different transformations? An obvious rationale would be steric shielding of either the *re*- or the *si*- face of the chiral catalyst-substrate adduct as the MacMillan group proposed.^101^ However, the mechanism of the CPA-catalyzed transfer hydrogenation was extensively studied by Gschwind^104–108^ and Goodman^109,110^ experimentally and computationally (Figure 14). The CPA catalyst and the *E*- or *Z*-imine form a binary hydrogen bond-assisted ion pair **35** followed by the binding of the Hantzsch ester. In this ternary adduct **37**, enantioselectivity arises from the bottom or top side directed hydride transfer of the Hantzsch ester to the *E-* or *Z*-imine providing four different transition structures.^104–108^ Computations indicate that the lowest energy TSs includes the *Z*-imine **34** and hydride transfer directed to the bottom side leading to the experimentally obtained enantioselectivities. Furthermore, the imine isomerization is slow compared to the hydride transfer, facilitating enantiocontrol of the reaction via increased population of the *Z*-imine. The Gschwind group found that the strategic positioning of *t*butyl groups as DEDs on the imine starting material led to a stabilization of the *Z*-isomer by around 1.0 kcal mol^–1^ and demonstrated that its preference is preserved in the binary and ternary CPA adducts.^111^ Counterintuitively, additional decoration of the CPA with intrinsically bulky DEDs did not lead to increased steric repulsion between catalyst and imine, rather, it added favorable LD interactions in the binding event of the catalyst and substrate leading to almost exclusive population of the desired *Z*-imine and excellent enantioselectivities.^111^ The List group revisited the transfer hydrogenation of enals^112–114^ identifying LD interactions as an important stereocontrolling factor.^115^ Impressively, DED-decorated TRIP-derived CPA catalysts continue to demonstrate broad applicability nearly two decades after their introduction.^102,103^

**Figure 14:**
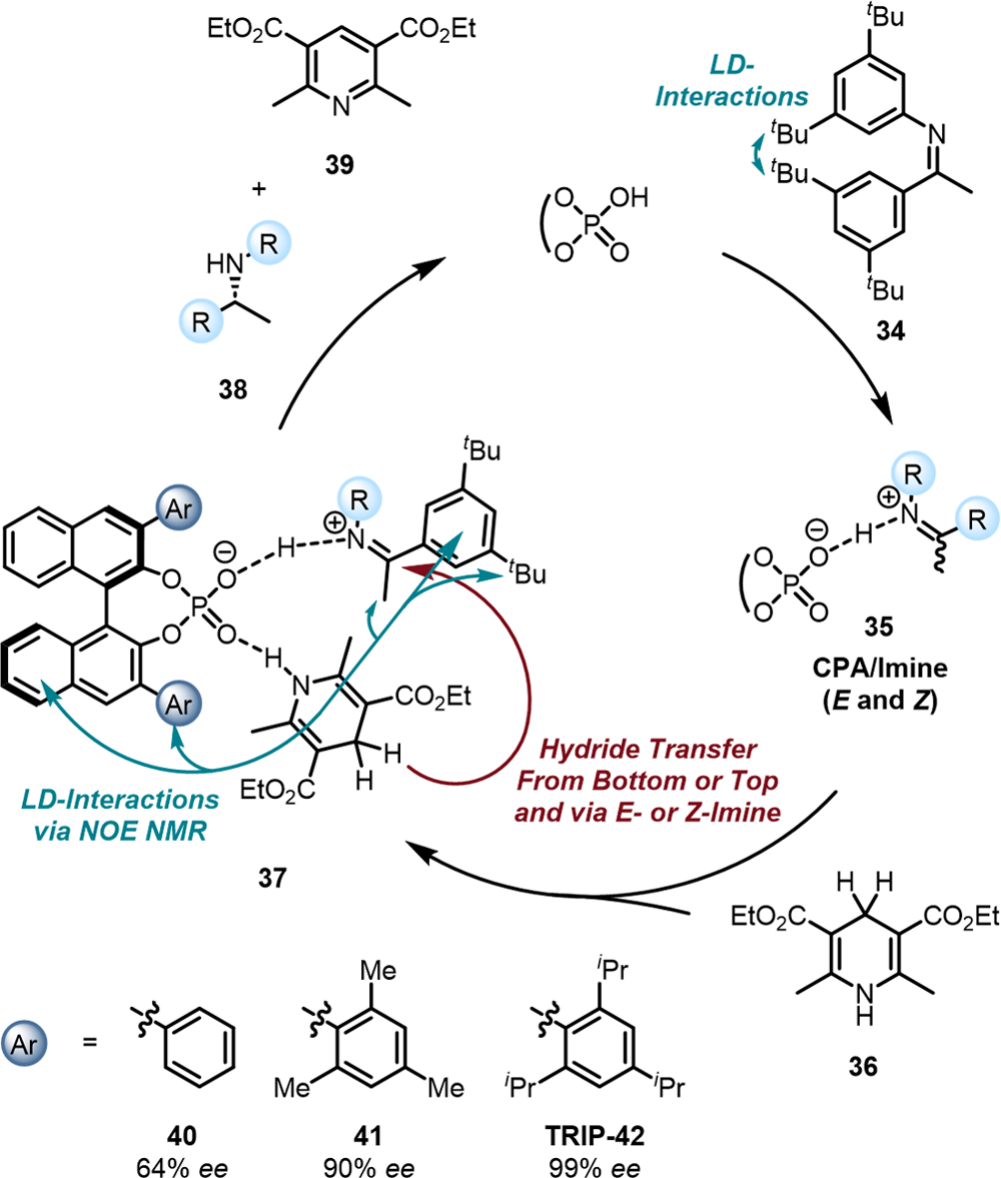
Mechanism of the CPA-catalyzed transfer hydrogenation of imines with Hantzsch esters.^111^

Further development of asymmetric counteranion-directed catalysis (ACDC)^103,116^ gave rise to more acidic and “confined” catalysts than CPAs (p*K*_a_ = 13.6 for TRIP in MeCN).^117^ Ultimately, the List group developed imidodiphosphorimidate (IDPi) catalysts which feature an acidic inner core (p*K*_a_ = 4.5 to ≤2.0 in MeCN)^117^ and two fused BINOL backbones surrounding it giving rise to narrow and highly confined, enzyme-like reaction pockets. This tremendous increase in acidity enabled various challenging organocatalytic transformations via Lewis- and Brønsted acid catalysis with excellent enantioselectivities guided by the substituents on the BINOL moieties. An impressive example of this type of chemistry is the IDPi catalyzed Diels–Alder (DA) reaction of unreactive *trans*-cinnamate esters with cyclopentadiene **44** (Figure 15).^118^ Here, the *in situ* silylated IDPi **46** forms a chiral ion pair (CIP) after transferring the silyl group to the ester moiety of **43**. This Lewis acid activation enables even unreactive *trans*-cinnamate esters to undergo cycloadditions with cyclopentadiene **44** even when methyl esters were employed that proved to be unreactive with other organocatalysts.^118–120^ Remarkably, the more confined IDPi catalysts are not only more selective than other BINOL-derived catalysts, they often also are more reactive even though the active site of the catalyst is sterically more congested. This is in contradiction with any model that builds on differential destabilization, which would reduce the rates, and points to a stabilizing and hence rate-enhancing interaction. Bistoni and co-workers used high-level computational methods [DLPNO–CCSD­(T)/def2TZVP] to identify stereocontrolling LD interactions guiding this challenging transformation.^120^ Counterintuitively, the steric bulk of the catalyst did *not* hamper its reactivity: the CIP structure can distort slightly to maximize LD in the relevant TSs^120^ reminiscent of the induced-fit model to describe enzyme activity.^121^ Therefore, the authors proposed that the implementation of appropriate DEDs to IDPi catalysts facilitates catalyst design.^120,121^

**Figure 15:**
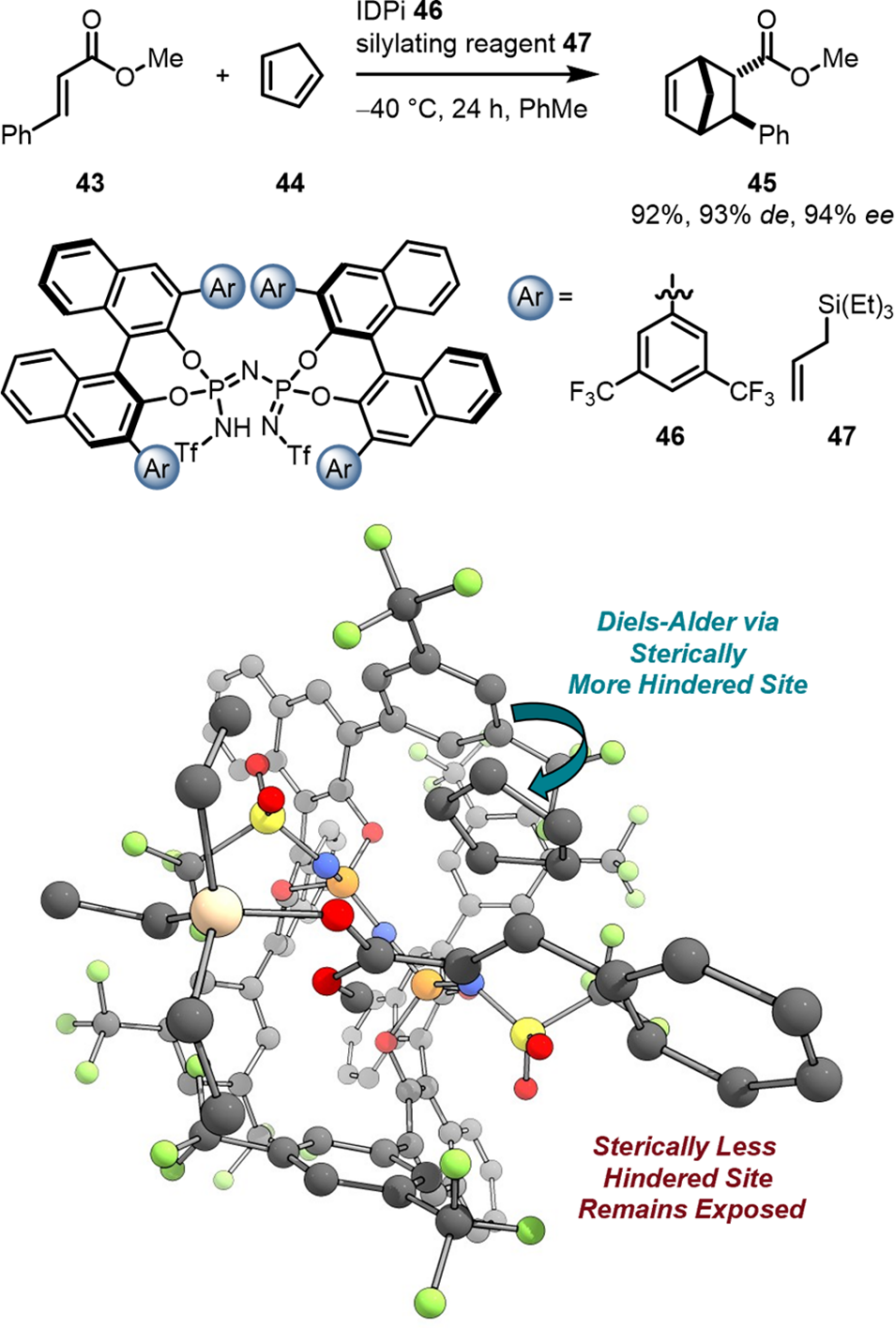
Transition structure for the IDPi catalyzed DA reaction of *trans*-cinnamate ester with cyclopentadiene.^120^ Hydrogens are omitted for clarity.

Small peptide catalysts take advantage of the strategic placement of DEDs into amino acid side chains, e.g., in the enantioselective kinetic resolution of *trans*-cyclohexane 1,2-diols.^122,123^ Here, cyclohexylalanine was incorporated in the peptide backbone to conformationally align the catalyst with the cyclohexyl moiety of the substrate via LD interactions. Computations as well as sophisticated 2D-NMR analyses indicate the formation of an enzyme-like pocket in which the substrate is bound via hydrogen bond- and LD interactions.^74,124^ Similar LD-controlled binding via attractive alky–alkyl contacts between cyclohexyl groups on catalyst and substrate was utilized in the enantioselective Dakin–West reaction (Figure 16).^73^ The oligopeptide **49** catalyzes the initial Steglich rearrangement followed by decarboxylation and enantioselective reprotonation of the enolate intermediates. The cyclohexyl group of the catalyst interacts favorably with DEDs of the substrate’s side chain to form more compact transition structures.^73^ These two examples demonstrate the relevance of LD interactions in organocatalysis with polar intermediates and hydrogen bonding interactions.^125,126^

**Figure 16:**
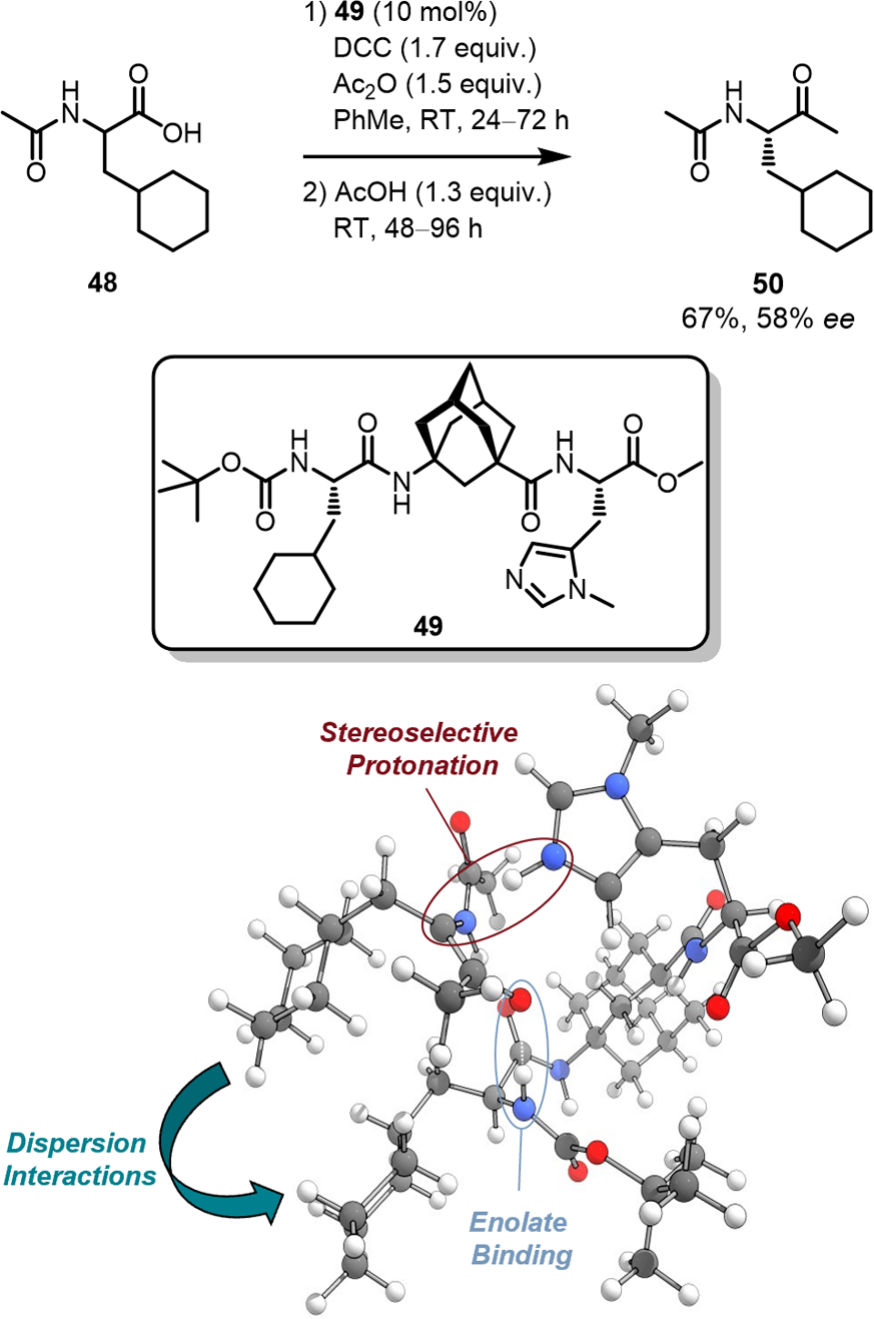
Stereoselective protonation step of the oligopeptide catalyzed Dakin–West reaction.

The presence of a charged metal center often led chemists to conclude that LD interactions are not relevant in transition metal chemistry. Usually, chemists refer to steric “through-space”^127^ models that describe NCIs between ligands and substrates or an electronic “through-bond” ^128,129^ mechanism that involves the metal center and the substrate.^130^ However, the through-space model fails to explain rate accelerations with more bulky ligands that have been reported in cross coupling reactions.^131–133^ For example, the Liu group^76^ demonstrated how *all* NCIs between substrate and ligands have to be taken into account to fully understand the Cu­(I) hydride-catalyzed hydroamination of unactivated olefins (Figure 17).^134^ Routinely employed ligands (e.g., SEGPHOS, BINAP) poorly catalyze the hydroamination of unactivated olefins providing the desired products in low yields. In contrast, when di-*t*butyl or di-*t*butyl-methoxy (DTBM) *P*-aryl substituents are employed on the same catalyst families’ reaction rates and yields increase drastically. Experimental and computational investigations of the rate-determining hydrocupration step revealed LD interactions to be the dominant contributor to lowering the activation barrier.^76^ Interestingly, the rate acceleration and the yields *increased* when 3,5-di-*t*butyl substituents were implemented on the *P*-aryl groups *regardless* of the phosphine ligand family indicating a catalytic generality to these catalysts.^76^ In fact, the DTBM substitution pattern excelled in a plethora of reactions, *e.g.*, in alkyne hydroalkylation^135^ and enantioselective olefin hydromethylations^136^ as demonstrated by Buchwald and co-workers.^137^

**Figure 17:**
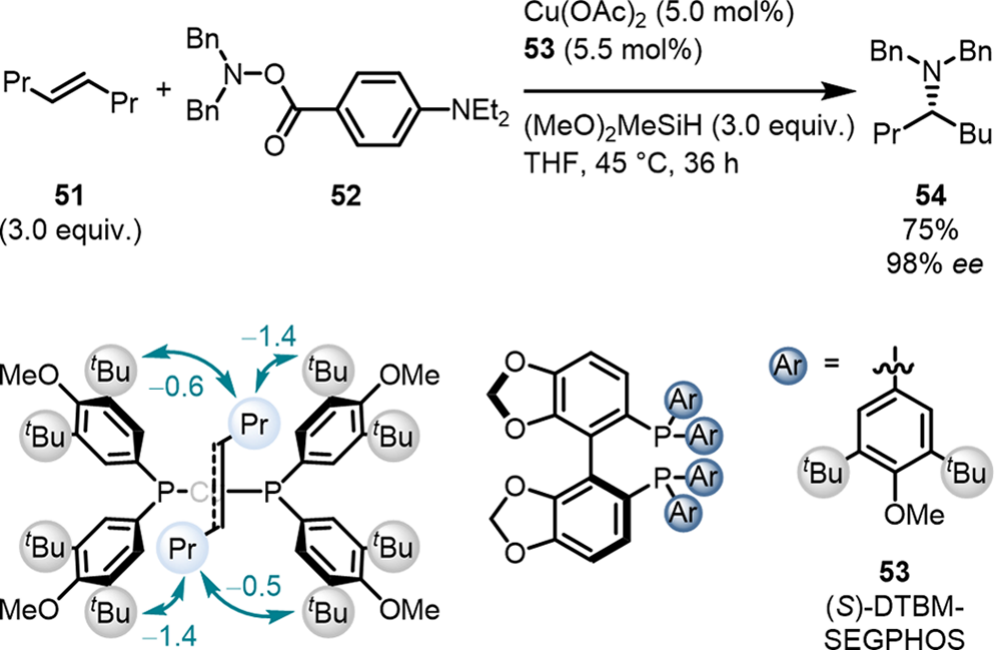
LD interactions between the propyl substituents of *trans*-4-octene and the *t*butyl moieties of the DTBM-SEGPHOS ligand in the hydrocupration TS. Energies (kcal mol^–1^) in teal represent the LD contribution.^76,134^

A similar catalyst generality can be assumed for the heterobimetallic bismuth–rhodium paddlewheel catalysts introduced by the Fürstner group enabling cyclopropanation, cyclopropenation as well as C–H and Si–H insertion reactions in a highly selective fashion (Figure 18).^138,139^ In these catalysts the commonly employed Rh_2_ center is replaced with unreactive Bi^2+^. Because of bismuth’s larger atomic radius, the calyx formed by the ligands around the active rhodium-site tightens leading to increased stereoselectivity.^140–142^ However, depending on the phthalimide and leucine substituents, the catalyst can form an inverted calyx in which the selective reaction pocket forms around bismuth and leaves the active rhodium-site exposed, thereby eroding selectivity.^142^ To gain control over the catalyst’s calyx directionality, TIPS and *t*butyl groups were installed on the leucine backbones of **56**.^138^ Intramolecular LD interactions between the TIPS and *t*butyl groups on the ligands stabilize the conformation and directionality of the formed catalytic pocket and renders it significantly more compact.^138^ Remarkably, the LD stabilization accounts for −11.6 kcal mol^–1^ with ca. 32% originating from favorable TIPS contacts and ca. 12% from the *t*butyl moieties.^138^ The increased confinement around the active site does *not* hamper the catalyst’s reactivity. Quite the opposite: the bulkier the catalyst the more active it is. Reaction times for the studied cyclopropanations reduced to 10 min at −10 °C from 3 h for the less crowded parent catalysts. Impressively, catalyst loading can be reduced to 0.005 mol % while still retaining feasible reaction times and almost perfect enantiocontrol.^138,139^

**Figure 18:**
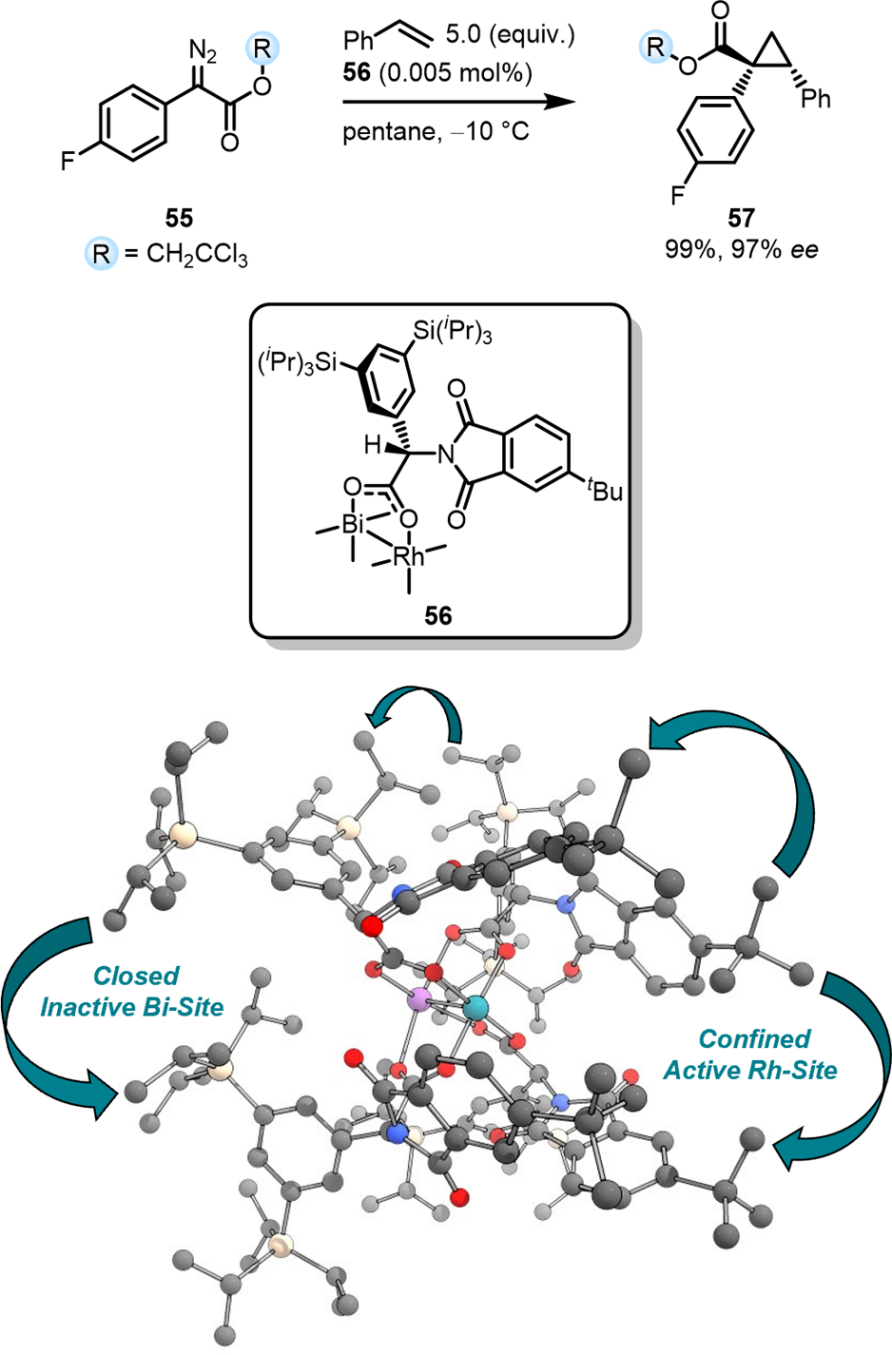
Cyclopropanation of styrene catalyzed by a LD stabilized heterobimetallic bismuth–rhodium paddlewheel complex. Geometry of the structure computed on PBE-D3­(BJ)/def2-TZVP level of theory.^138^ Hydrogens are omitted for clarity.

## Concluding Remarks

This perspective aims to introduce London dispersion (LD) as a physically well-grounded concept to a broader audience in the field of catalysis. Beginning with its theoretical foundation, we discuss the fundamentals of LD, its antagonism to steric repulsion, its role in molecular recognition, and how molecular balances can be employed to quantify its relevance. LD persists (to different degrees) in all aggregation states and thus significantly affects reaction outcomes. The concept of steric attraction (through LD) emerges as a powerful tool in modern synthetic (organic) chemistry, as the traditional model of steric hindrance is increasingly being broadly recognized as insufficient to fully explain stereochemical outcomes in catalyzed reactions.

Harnessing LD provides a more nuanced understanding of molecular interactions, with the potential to advance catalyst design and reaction development. LD may be even more important in biological systems such as enzymes.^143^ Early enzyme models relied on steric repulsion to explain substrate discrimination (e.g., the lock-and-key principle),^144^ which later evolved into the more sophisticated induced-fit model^145^ that emphasizes dynamic substrate–enzyme interactions.

As an outlook, we remark that LD may also be highly relevant for photoexcited states for which van der Waals volumes increase, leading to higher polarizabilities and hence increased LD interactions.^146,149^
